# A survey of the workplace experiences of police force employees who are autistic and/or have attention deficit hyperactivity disorder

**DOI:** 10.1192/bjo.2023.508

**Published:** 2023-07-06

**Authors:** Samuel J. Tromans, Alison Drewett, Paul H. Lee, Michelle O'Reilly

**Affiliations:** Department of Population Health Sciences, University of Leicester, UK; and Adult Learning Disability Service, Leicestershire Partnership NHS Trust, UK; School of Design and Creative Arts, Loughborough University, UK; and Speech and Language Therapy Service, Leicestershire Partnership NHS Trust, UK; Department of Population Health Sciences, University of Leicester, UK; and Southampton Clinical Trials Unit, University of Southampton, UK; School of Media, Communication and Sociology and School of Psychology and Vision Sciences, University of Leicester, UK; and Families, Young People and Children's Service, and Learning Disabilities, Leicestershire Partnership NHS Trust, UK

**Keywords:** Autism spectrum disorder, attention-deficit hyperactivity disorder, comorbidity, stigma and discrimination, developmental disorder

## Abstract

**Background:**

There has been little focus on autism and attention-deficit hyperactivity disorder (ADHD) in occupational groups, particularly in high-demand roles such as the police.

**Aims:**

To describe the characteristics and experiences of UK-based police force employees who are autistic and/or have ADHD, including the benefits and challenges their conditions bring to their occupation, their need for reasonable adjustments, and their co-occurring mental illnesses.

**Method:**

An online survey was developed, containing both quantitative and qualitative elements. Survey invitations were disseminated through the National Police Autism Association. The survey was open from 23 April to 23 July 2022.

**Results:**

A total of 117 participants participated in the survey, including 66 who were autistic and 51 with ADHD. Participants who were autistic and/or had ADHD widely reported both benefits and challenges related to their condition(s) in policing work. Both the autistic and ADHD groups widely reported having requested workplace adjustments related to their condition(s), although these were frequently not made. Anxiety (*n =* 57; 49%) and depression (*n =* 40; 36%) were both highly prevalent among the participants.

The qualitative findings identified four themes: (a) motivations for taking on this career, (b) rewards of the role, (c) challenges of the job and (d) challenges regarding career progression.

**Conclusions:**

Police force employees who are autistic and/or have ADHD reported that their conditions provided both benefits and challenges with respect to policing work, and that they had requested related workplace adjustments, although such adjustments frequently do not take place. Healthcare professionals need to recognise the importance of workplace considerations and advocacy for people who are autistic and/or have ADHD.

Autism spectrum disorder (hereafter referred to as autism) is described by the DSM-5^1^ as being characterised by ‘persistent deficits in social communication and social interaction across multiple contexts’ as well as presence of ‘restricted, repetitive patterns of behavior, interests, or activities.’ Approximately 1% of adults are autistic,^2^ and autistic persons experience a significantly greater burden of mental illness compared with their non-autistic peers.^3^ Attention-deficit hyperactivity disorder (ADHD) is characterised by ‘impairing levels of inattention, disorganization, and/or hyperactivity-impulsivity.’^1^ A meta-analysis by Song et al^4^ estimates prevalence rates of 2.58% and 6.76% for persistent adult ADHD (with childhood onset) and symptomatic adult ADHD (irrespective of childhood onset), respectively. ADHD similarly appears to be associated with increased risks of mental and physical illness.^5^ In contrast to autism,^6^ ADHD is overrepresented within the criminal justice system.^7^ Furthermore, there is substantial underemployment among adults who are autistic^8^ and/or have ADHD^9^ compared with their peers. Demographic factors also influence people's experiences of both conditions, including sex and gender,^10^ age,^11,12^ and whether they have a clinical diagnosis of autism and or ADHD^13^ (in contrast with individuals who self-identify as autistic and/or as having ADHD but lack a clinical diagnosis).

Previous research pertaining to autism and ADHD and the police has largely focused on where members of the public who are autistic and/or have ADHD have had encounters with the police, including their experiences,^14^ their prevalence within the criminal justice system^6,15^ and police training interventions in understanding these conditions.^16^ However, there has been comparatively little research into police employees who are themselves autistic and/or have ADHD, despite these individuals requiring support as well as potentially having abilities that offer significant benefits to police work, such as those relating to pattern recognition,^17^ detail-focused processing, memory^18^ and divergent (lateral) thinking.^19^

Research more generally into the workplace experiences of autistic adults describes positive factors, such as self-actualisation, enjoyable interactions with colleagues and clients, and earning money, as well as negative aspects including difficulties communicating with others, poor working conditions and challenges to sensory sensitivities in the workplace.^20^ Considering the importance of effective communication in police work, as well as the frequently challenging environments police force employees work in, such difficulties may have a particular impact in this occupational group. By contrast, prior research into the workplace experiences of people with ADHD reported that they expressed concerns about not meeting their perceived potential in the workplace, and inattention symptoms demonstrated a strong association with work-related problems.^21^ Police work can involve situations where maintenance of attention is critical to the safety of both self and others, so such problems may be more manifest in people with ADHD working in the police force.

In this study, we conducted an online survey of UK-based police force employees to describe the characteristics and workplace experiences of those who were autistic and/or had ADHD, including the benefits and challenges their conditions bring to their working life, their need for reasonable adjustments and the organisational response to such needs, and rates of co-occurring mental illness. We also conducted subgroup analyses concerning other factors that were likely to influence workplace experiences, including gender, age and length of employment, as well as having a clinical diagnosis of autism and/or ADHD.

## Method

A survey was developed (see Supplementary information available at https://doi.org/10.1192/bjo.2023.508) by three of the authors to ascertain the workplace experiences of police employees who were autistic and/or had ADHD. The survey contained both quantitative and qualitative elements. The survey included questions related to the benefits or challenges of individuals’ autistic and/or ADHD features relating to their policing work, awareness of their colleagues of their diagnosis or diagnoses, how well supported they felt, their own understanding of their needs, their colleagues’ and the general public's understanding of autism and/or ADHD, whether they would recommend a career in police work for an adult who is autistic and/or has ADHD and whether their career had progressed in the way they wanted. Additional sections of the questionnaire related to participants’ basic demographic details, as well as any co-occurring mental and or physical health conditions, and whether they took any medications for such conditions.

The survey was transferred to an online survey platform,^22^ and survey invitations were sent via e-mail to members of the National Police Autism Association^23^ (NPAA)^a^ and UK-based police forces, as well as being advertised in the NPAA newsletter. As of 17 March 2022,^b^ the NPAA had 1984 members, compared with 140 228 full-time equivalent police force employees based in England and Wales as of 31 March 2022.^24^

The first page of the online survey comprised a consent form, with a box that the participant ticked to confirm their consent to take part in the study. The survey was available from 23 April 2022 to 23 July 2022.

With respect to the positionality of the authors, one of the authors has a clinical diagnosis of ADHD. Three of the authors have first-degree relatives with the conditions under study in this survey, one with an autistic sibling, another with a sibling who is autistic with co-occurring ADHD and another with an autistic child. Two of the authors have clinical experience of working with people who are autistic and/or have ADHD. None of the study authors have personal experience of working in the police force or having close family members working in the police force. The research team developed the study following liaison with a senior member of the NPAA.

### Ethical approval

Ethical approval for the study was obtained from the University of Leicester Ethics Service (approval code: 32020-mjo14-ss/mc:sociology).

### Analysis

#### Quantitative analysis

Quantitative analyses were completed in IBM SPSS version 28. Fisher's exact test was used for significance testing, with the threshold for statistical significance set at *P <* 0.05. The autistic group was defined as those who responded with ‘yes’ to the item ‘Do you believe that you are autistic and/or have a clinical diagnosis of autism, made by a qualified healthcare professional?’, and the ADHD group was defined as those who responded with ‘yes’ to the equivalent question for ADHD. This approach enabled recognition of adults who self-identified as being autistic and/or having ADHD but lacked a clinical diagnosis. There are barriers to accessing a clinical diagnosis in the UK for both conditions, including a lack of awareness of the conditions among many healthcare professionals, as well as lengthy waiting lists for diagnostic assessment.^25,26^ Within the autistic and ADHD groups, subgroup data are reported according to sex (male or female), year of birth (<1980 or ≥1980),^c^ year joining the police force (<2006 or ≥2006)^d^ and presence of clinical diagnosis (clinical diagnosis or self-diagnosis only).

#### Qualitative analysis

The qualitative survey data were explored using a codebook type of thematic analysis, as this allowed for a conflation of deductive coding based on the quantitative findings, and inductive interrogation of text provided by participants.^27^ Braun and Clarke^27^ demonstrated that the codebook approach to thematic analysis allows analysts to develop the final codebook as influenced by engagement with the literature and can also be participant-centred through a familiarisation process. Consistent with this approach, we recognised how our questions shaped some of the responses provided given the nature of the survey, but two coders explored the data independently and then mapped the final codebook to ensure researcher agreement and consistency in code and theme labels.

## Results

### Characteristics of participant population

A total of 117 individuals participated in the study, taking a mean average of 19 min to complete the survey (range 1–175 min, s.d. 25 min). The characteristics of the participant population are summarised in Table 1, subdivided into four groups: autistic but not ADHD (*n =* 49); ADHD but not autistic (*n =* 34); both autistic and ADHD (*n =* 17); and neither autistic nor ADHD (*n =* 17). In terms of gender,^28^^e^ the participants comprised 54 men (46%), 58 women (50%) and three non-binary and/or non-conforming persons (3%). Participants who were neither autistic nor had ADHD were more likely to be female (83%), and the gender ratios were close to 1:1 in the other three groups. Most survey participants reported being 31–50 years of age (*n =* 81; 69%), with 11 participants (9%) not providing data on their age. With respect to ethnicity, most participants reported being of White British or Northern Irish ethnicity (*n =* 106; 91%), with 8% of participants (*n* = 9) reporting membership of other ethnic groups and 2% (*n =* 2) not providing data pertaining to ethnic group membership. More than three-quarters of the participant population joined the police force in 2003 or later (*n =* 89; 76%). The distributions of ethnicity and year joining the police force were similar in the four groups.

**Table 1:** Demographics of the study population (*n* = 117)

Variable	Category	Frequency, *n* (percentage)				
		Overall (*n* = 117)	Autistic but not ADHD (*n* = 49)	ADHD but not autistic (*n* = 34)	Both autistic and ADHD (*n* = 17)	Neither autistic nor ADHD (*n* = 17)
Gender	Male	54 (46%)	27 (55%)	15 (44%)	6 (35%)	6 (35%)
	Female	58 (50%)	20 (41%)	17 (50%)	10 (59%)	11 (65%)
	Non-binary/non-conforming	3 (%)	1 (2%)	1 (3%)	1 (6%)	0 (0%)
	Prefer not to say	1 (1%)	4 (8%)	0 (0%)	0 (0%)	0 (0%)
	Missing	1 (1%)	0 (0%)	1 (3%)	0 (0%)	0 (0%)
Age, years	30 or younger	11 (9%)	4 (8%)	3 (9%)	3 (18%)	1 (6%)
	31–40	28 (24%)	12 (25%)	8 (24%)	4 (24%)	4 (24%)
	41–50	53 (45%)	22 (45%)	15 (44%)	7 (41%)	9 (53%)
	51 or older	14 (12%)	8 (16%)	1 (3%)	2 (12%)	3 (18%)
	Missing	11 (9%)	3 (6%)	7 (21%)	1 (6%)	0 (0%)
Ethnicity	White (English/Welsh/Scottish/Northern Irish/British)	106 (91%)	46 (94%)	30 (88%)	16 (94%)	14 (82%)
	Others	9 (8%)	2 (4%)	3 (9%)	1 (6%)	3 (18%)
	Missing	2 (2%)	1 (2%)	1 (3%)	0 (0%)	0 (0%)
Year joining the police force	After 2013	40 (34%)	11 (32%)	11 (32%)	8 (47%)	5 (29%)
	2003–2012	49 (42%)	18 (37%)	18 (53%)	5 (29%)	8 (47%)
	1993–2002	21 (18%)	2 (6%)	2 (6%)	4 (24%)	4 (24%)
	On or before 1992	1 (1%)	0 (0%)	0 (0%)	0 (0%)	0 (0%)
	Missing	6 (5%)	3 (9%)	3 (9%)	0 (0%)	0 (0%)
Autism	Yes, with clinical diagnosis	44 (38%)	32 (65%)	0 (0%)	12 (71%)	0 (0%)
	Yes, without clinical diagnosis	22 (19%)	17 (35%)	0 (0%)	5 (29%)	0 (0%)
	No	42 (36%)	0 (0%)	30 (880%)	0 (0%)	12 (70%)
	Unsure	8 (7%)	0 (0%)	4 (12%)	0 (0%)	4 (24%)
	Missing	1 (1%)	0 (0%)	0 (0%)	0 (0%)	1 (6%)
ADHD	Yes, with clinical diagnosis	30 (26%)	0 (0%)	24 (71%)	6 (35%)	0 (0%)
	Yes, without clinical diagnosis	21 (18%)	0 (0%)	10 (29%)	11 (65%)	0 (0%)
	No	57 (49%)	42 (86%)	0 (0%)	0 (0%)	15 (88%)
	Unsure	9 (8%)	7 (14%)	0 (0%)	0 (0%)	2 (12%)
Co-occurring mental health conditions	Depression	40 (34%)	16 (33%)	13 (38%)	5 (29%)	6 (35%)
	Anxiety	57 (49%)	24 (49%)	16 (47%)	7 (41%)	7 (59%)
	Post-traumatic stress disorder	9 (5%)	3 (6%)	1 (3%)	2 (12%)	0 (0%)
	Low mood	3 (3%)	2 (4%)	1 (3%)	0 (0%)	0 (0%)
Taking medication for mental health	Antidepressant	38 (32%)	16 (33%)	12 (35%)	4 (24%)	6 (35%)
	Anti-anxiety	4 (3%)	2 (4%)	1 (3%)	0 (0%)	1 (6%)
	Antipsychotic	2 (2%)	0 (0%)	2 (6%)	0 (0%)	0 (0%)
	Mood stabiliser	1 (1%)	0 (0%)	1 (3%)	0 (0%)	0 (0%)
Co-occurring physical health conditions	Sleep apnoea	2 (2%)	1 (2%)	1 (3%)	0 (0%)	0 (0%)
	Gastrointestinal disease	8 (7%)	3 (6%)	4 (12%)	1 (6%)	0 (0%)
	Cardiovascular disease	4 (3%)	2 (4%)	1 (3%)	1 (6%)	0 (0%)
	Musculoskeletal problems	19 (16%)	10 (20%)	3 (9%)	3 (18%)	3 (18%)
	Diabetes/pre-diabetes	5 (4%)	2 (4%)	1 (3%)	1 (6%)	1 (6%)
	Asthma	8 (7%)	4 (8%)	3 (9%)	1 (6%)	0 (0%)

ADHD, attention-deficit hyperactivity disorder.

Of the participant population, 66 (56%) reported believing themselves to be autistic and/or having a clinical diagnosis of autism made by a qualified healthcare professional, with 44 of these individuals (67% of the autistic subgroup) having a clinical diagnosis. By comparison, 51 (44%) reported believing themselves to have ADHD and/or having a clinical diagnosis of ADHD made by a qualified healthcare professional, with 30 of these participants (59% of the ADHD subgroup) having a clinical diagnosis.

With respect to mental health conditions, anxiety-related conditions were highly prevalent, with 57 (49%) participants reporting anxiety and nine (5%) reporting post-traumatic stress disorder. Depression was also highly prevalent, being reported by 40 (34%) participants, with a further three (3%) participants reporting low mood. No participant reported having bipolar disorder or schizophrenia. Many participants reported taking prescribed medications for their mental health, including 38 (32%) taking antidepressants, four (3%) taking anti-anxiety medication, two (2%) taking antipsychotic medication and one (1%) taking a mood stabiliser.

With respect to physical health conditions, musculoskeletal-related problems were the most prevalent, being reported by 19 (16%) of participants, followed by gastrointestinal conditions (*n* = 8; 7%) and asthma (*n =* 8; 7%).

### Quantitative findings

#### Autistic participants

A summary of the findings from autistic participants (*n =* 66) is shown in Table 2. However, please note that some autistic participants opted not to provide answers for all the survey questions; in this section, we refer to ‘participants’ when describing the findings of participants who provided responses to the survey item in question.

**Table 2:** Questionnaire responses for participants identifying as autistic (*n* = 66). The data reported in the table relate to ‘yes’ answers to the corresponding questionnaire item

Questionnaire item	Overall	Missing/unsure^a^	Gender			Year of birth			Year joining the police force			Clinical diagnosis (autism)		
			Male	Female	*P*	<1980	≥1980	*P*	<2006	≥2006	*P*	Yes	No	*P*
Do you believe that your autistic features provide benefits to you with respect to your policing work?	43 (81%)	13	22 (85%)	19 (79%)	0.72	22 (85%)	19 (83%)	0.99	25 (89%)	16 (73%)	0.16	31 (91%)	12 (63%)	0.025
Do you believe that your autistic features provide challenges to you with respect to your policing work?	59 (89%)	3	22 (88%)	27 (90%)	0.99	28 (90%)	28 (90%)	0.36	33 (97%)	23 (79%)	0.04	41 (93%)	18 (82%)	0.29
In your view, do you require adjustments to your role at work due to your autism diagnosis?	43 (68%)	14	18 (55%)	25 (83%)	0.03	22 (71%)	20 (65%)	0.87	23 (68%)	19 (66%)	0.75	32 (73%)	11 (50%)	0.18
Have you or someone on your behalf requested adjustments to my role at work due to your autism diagnosis?	38 (58%)	2	18 (55%)	20 (67%)	0.71	20 (65%)	17 (55%)	0.43	19 (56%)	18 (62%)	0.80	28 (64%)	10 (46%)	0.31
(If ‘yes’ to above item) Were these requested adjustments made?	22 (55%)	6	12 (63%)	10 (48%)	0.69	11 (52%)	10 (59%)	0.12	9 (45%)	12 (63%)	<0.001	18 (62%)	4 (36%)	0.057

a. Denotes participants selecting an ‘unsure’ response to the question item, or not providing a response.

With respect to whether they believed that their autistic features provided benefits to them with respect to policing work, 81% (*n =* 43) of participants said ‘yes.’ No significant differences in response were observed between males and females (*P =* 0.72), in those born prior to 1980 compared with those born during or after 1980 (*P =* 0.99), or in those who joined the police force prior to 2006 compared with those who joined during or after 2006 (*P =* 0.16). However, participants with a clinical diagnosis of autism were significantly more likely (*P =* 0.025) to report their autistic features providing such benefits to them (*n =* 31; 91%) compared with their peers without a clinical diagnosis (*n* = 12). Conversely, most participants also reported that their autistic features provided challenges to them with respect to their policing work (*n =* 59; 89%). Participants who had joined the police force prior to 2006 were significantly more likely to report such challenges relative to their peers who had joined during or after 2006 (*P =* 0.04).

More than two-thirds of participants (*n =* 43; 68%) reported viewing themselves as requiring adjustments to their role at work owing to their autism diagnosis; women were significantly more likely to view themselves as requiring adjustments compared with their male peers (*P =* 0.03). More than half of participants (*n =* 38; 58%) reported that they or someone on their behalf had requested adjustments to their role at work owing to their autism diagnosis, with no significant differences between the subgroups under comparison (gender: *P* = 0.71; year of birth (prior to or after 1980): *P =* 0.43; year joining the police force (prior to or after 2006): *P =* 0.80; clinical diagnosis of autism: *P =* 0.31). In terms of whether requested adjustments were made, 55% of participants (*n =* 22) reported that this had happened, although those who had joined the police force prior to 2006 were significantly less likely to report adjustments being made (*P <* 0.001).

### Participants with ADHD

A summary of the findings from participants with ADHD (*n =* 51) are shown in Table 3. However, as for the autistic participants, please note that some participants with ADHD opted not to provide answers for all the survey questions; in this section, we refer to ‘participants’ when describing the findings of participants who provided responses to the survey item in question.

**Table 3:** Questionnaire responses for participants identifying as having ADHD (*n* = 51). The data reported in the table relate to ‘yes’ answers to the corresponding questionnaire item.

Questionnaire item	Overall	Missing/unsure	Gender			Year of birth			Year joining the police force			Clinical diagnosis (ADHD)		
			Male	Female	*P*	<1980	≥1980	*P*	<2006	≥2006	*P*	Yes	No	*P*
Do you believe that your ADHD symptoms provide benefits to you with respect to your policing work?	35 (78%)	5	16 (80%)	18 (75%)	0.73	13 (93%)	19 (73%)	0.22	16 (100%)	18 (67%)	0.02	24 (86%)	11 (65%)	0.14
Do you believe that your ADHD symptoms provide challenges to you with respect to your policing work?	49 (98%)	1	21 (100%)	27 (100%)	N/A	16 (100%)	27 (100%)	N/A	17 (94%)	30 (100%)	0.38	30 (100%)	19 (95%)	0.40
In your view, you require adjustments to your role at work due to your ADHD diagnosis?	36 (72%)	9	17 (81%)	19 (70%)	0.61	13 (81%)	19 (70%)	0.87	16 (89%)	19 (63%)	0.19	25 (83%)	11 (55%)	0.03
Have you or someone on your behalf requested adjustments to my role at work due to your ADHD diagnosis?	23 (51%)	5	11 (58%)	11 (44%)	0.54	8 (53%)	13 (57%)	0.99	11 (65%)	12 (46%)	0.35	17 (63%)	6 (33%)	0.07
(If ‘yes’ to above item) Were these requested adjustments made?	8 (35%)	4	6 (55%)	2 (18%)	0.25	3 (38%)	3 (23%)	0.72	4 (36%)	4 (33%)	0.11	7 (41%)	1 (17%)	0.70

ADHD, attention-deficit hyperactivity disorder.

With respect to whether they believed that their ADHD features provided benefits to them with respect to policing work, 78% (*n =* 35) of participants said ‘yes.’ Participants who had joined the police force prior to 2006 were significantly more likely (*P =* 0.02) to report their ADHD features providing such benefits to them. However, most participants also reported that their ADHD features provided challenges to them with respect to their policing work (*n =* 49; 98%).

Almost three-quarters of participants (*n =* 36; 72%) reported viewing themselves as requiring adjustments to their role at work owing to their ADHD diagnosis; participants with a clinical diagnosis of ADHD were significantly more likely to view themselves as requiring adjustments compared with their peers with a self-diagnosis (*P =* 0.03). Approximately half of participants (*n =* 23; 51%) reported that they or someone on their behalf had requested adjustments to their role at work owing to their ADHD diagnosis, with no significant differences between the subgroups under comparison (gender: *P* = 0.54; year of birth (prior to or after 1980): *P =* 0.99; year joining the police force (prior to or after 2006): *P =* 0.35; clinical diagnosis of ADHD: *P =* 0.07). In terms of whether requested adjustments were made, 35% of participants (*n =* 8) reported that this had happened, with no significant differences between the same subgroups (gender: *P* = 0.25; year of birth (prior to or after 1980): *P =* 0.72; year joining the police force (prior to or after 2006): *P =* 0.11; clinical diagnosis of ADHD: *P =* 0.70).

### Associations with self-reported mental health conditions

Table 4 shows the associations of self-reported depression and anxiety with pertinent demographic and clinical factors. Among the participant population, high rates of both anxiety (49%; *n =* 57) and depression (34%; *n =* 40) were reported. No statistically significant differences were found between the previously described demographic subgroups and either self-reported depression (gender: *P* = 0.69; year of birth (prior to or after 1980): *P =* 0.42; year joining the police force (prior to or after 2006): *P =* 0.33) or anxiety (gender: *P* = 0.19; year of birth (prior to or after 1980): *P =* 0.99; year joining the police force (prior to or after 2006): *P =* 0.71). Furthermore, there were no statistically significant associations between having a clinical diagnosis of autism or ADHD and self-reported depression (autism: *P =* 0.54; ADHD: *P =* 0.84) or anxiety (autism: *P =* 0.84; ADHD: *P =* 0.70).

**Table 4:** Questionnaire responses from the study population (*n* = 117) relating to self-reported depression and anxiety, in response to the questionnaire item ‘Please list your mental health conditions’

Mental health condition	Overall	Missing/unsure	Gender			Year of birth			Year joining the police force			Clinical diagnosis (autism)			Clinical diagnosis (ADHD)		
			Male	Female	*P*	<1980	≥1980	*P*	<2006	≥2006	*P*	Yes	No	*P*	Yes	No	*P*
Depression	40 (34%)	0	18 (33%)	22 (38%)	0.69	15 (30%)	22 (39%)	0.42	18 (32%)	22 (41%)	0.33	21 (32%)	16 (38%)	0.54	18 (35%)	18 (32%)	0.84
Anxiety	57 (49%)	0	23 (43%)	33 (57%)	0.19	25 (50%)	29 (52%)	0.99	28 (49%)	29 (54%)	0.71	31 (47%)	21 (50%)	0.84	23 (45%)	28 (49%)	0.70

ADHD, attention-deficit hyperactivity disorder.

### Qualitative findings

The qualitative coding process led to the generation of four central themes related to the benefits and challenges of being a person who is autistic and/or has ADHD working for the police; while presenting slightly differently in some cases, these were mostly consistent for autism and ADHD. These themes were: (a) motivations for taking on this career, (b) rewards of the role, (c) challenges of the job and (d) challenges of career progression. Working for the police force can be a complex, stressful and demanding career, and thus the characteristics associated with autism and/or ADHD were discussed in relation to this job profile. Notably, the four themes were not represented equally in depth, breadth or meaning; although we report all four themes here, we note that the challenges of working for the police as a person who is autistic and/or has ADHD represented by far the most significant theme. This is consistent with studies of autistic employees’ experiences of the workplace more generally, which have found overwhelmingly negative reports and workplace stress.^29^

### Theme one: motivations for career choice

Participants reported motivations for a career in the police that in some ways reflected the characteristics associated with their autism and/or ADHD. For example, autistic persons alluded to the value of structure and job security, whereas those with ADHD considered diversity in the role to be a motivation for their choice.

*It kind of just went from there – structure, guarantee of work/pay and (at the time) pretty good benefits*.

*Wanted to do the job as a child. Like structure and discipline*.

*Doing something different every day*.

Many of the participants cited helping others as the primary driver for their career direction; this was the case for both autism and ADHD. They discussed the different ways in which they felt a career in the police would enable them to help other people, and this was posited as important to them and to their day-to-day lives in the job. They wanted work that was meaningful and was exciting, and they wanted to do something where they could help people.

*Meaningful work – as it works to protect the community, exciting role,*

*I really felt it was a role where I could help people*.

### Theme two: rewards of the role

The participants in this study were clear that there were rewards within the police force that helped to maintain a drive to undertake the work. Mostly, this related to a narrative of justice, contributing to society and serving victims of crime.

*Ensuring a thorough investigation in completed (at least on my part) to assist victims of crime*.

*I enjoy knowing that I have done my best for the victim*.

*Knowing I am contributing to a job and helping bring justice to families of victims*.

A central discourse for the participants involved highlighting their commitment to the victims of crime as they positioned themselves as wanting to do their best to help them.

Aligned with justice was a discourse of ensuring that they had a role in managing criminal behaviour and played a central part in ensuring that criminals were no longer able to continue with their unlawful acts, which they constructing as ‘catching criminals’.

*Catching burglars, sex offenders and the most violent people*.

*Catching criminals and putting them before a court*.

The participants recognised that to help victims and fulfil the objectives of their policing role, they needed to attend to the criminals and to ensure that these cases reached court.

Notably, there was also, for some, an individualistic sense of reward as they reported that the role within the police force brought personal benefits, such as skills development and being challenged.

*Getting to engage and reassure members of public and being able to deal with lots of different things every day all at once*.

*I feel it stretches me and utilises my skills thoroughly*.

*I see people at their very best and also at their very worst – it's both rewarding and highly challenging, but I love to be challenged and find it a very grounding position to be in*.

### Theme three: challenges of the role

The participants highlighted a range of challenges relating to working for the police, and although some challenges were reported as direct consequences of their autism and/or ADHD, other challenges related more to wider structural, organisational and systemic issues within the public sector. These wider challenges were nonetheless positioned within a neurodevelopmental profile of the individual and thus related to their perceptions of their own condition and how they believed others saw them.

Participants highlighted that there were features of their autism and/or ADHD that they felt had an impact on their daily work lives, particularly in relation to social interactions with other members of the police force.

*Not burning out, my ADHD wears me out severely, so getting sufficient rest mentally from the intensity ADHD in the job brings in my head*.

*Socialising and making friends. I have always struggled with these, particularly with females around my own age*.

Here, there were instances where participants recognised their individual limitations or difficulties, as the characteristics of their condition made things more difficult for them. However, there were also challenges caused by the organisational response and the views or behaviour of others. In many cases, they did not disclose their neurodevelopmental condition to their peers or managers because of the risks they considered this to entail, and indeed this was confirmed for some.

*Lack of knowledge around ADHD and my feeling unable to explain this to my previous managers has meant I have deliberately not moved to roles I would like for fear of there being no support for me to learn new skills*.

*I believe I am perceived as being a bit ‘strange’ and it is this that has prevent previous opportunities*.

*Been held back by disclosing autism diagnosis*.

According to the data, challenges of the workplace were thought to result in discrimination and burnout, which can risk poorer mental health outcomes for those individuals.

*Being misunderstood, dealing with discrimination and challenged culture*.

*I have then reached a burnout phase in both, leading to a skill drop which caused me to feel pushed out of both roles. Due to the hostile and sexist attitude in both of these areas, any issues make you feel extremely vulnerable*.

As noted, working for the police can be a stressful and demanding occupation, and these participants recognised that such issues were exacerbated by dealing with discrimination and a hostile and sexist attitude, which could lead to burnout and feelings of vulnerability.

Organisational factors were also highlighted as problematic, with both autistic participants and those with ADHD citing problems with workload, bureaucracy (framed as ‘red tape’) and poor leadership.

*Dealing with red tape and constantly jumping through hoops*.

*Dealing with management, the role is emotionally draining and physically demanding but a job like no other, ruined by weak management and poor decision making*.

*The volume of workload is not sustainable and means we give victims a lesser service than I would like, and this caused me stress*.

### Theme four: career progression

The fundamental message that participants were keen to convey through the qualitative aspect of the questionnaire was the detrimental impact of the neurodevelopmental condition on their career pathway. Many of the participants (both autistic and ADHD) reported that their characteristics had created challenges to promotion and progression. This was related to processes and structures, lack of reasonable adjustments and difficulties in implementation when they were provided, lack of knowledge and understanding by senior leaders, and a range of barriers within the organisation.

*I believe my ADHD has held me back*

*ADHD has definitely made it more difficult to progress as my ADHD makes it very difficult to say the right thing to line managers and job specific conversations to aid promotion/transfer to specialised teams*.

Especially noteworthy in the responses was that despite reasonable adjustments where they were recognised or asked for, there were still challenges in terms of career progression and promotion.

*I have asked for reasonable adjustment in interview it didn't go well. I took a prompt card into an interview with the agreement of HR on the interview was said they thought it was cheating I didn't get the job*.

*The frustration at knowing that I am junior in rank, feel capable of operating at a far higher level but as I am not a networker, am not appreciated, am overlooked I do not get support/encouragement to help me. On top of this the promotion processes are set up in a way which disadvantages me*.

## Discussion

This article reports survey responses from UK-based individuals working for the police force, with a focus on their experiences of being autistic and/or having ADHD while being a police force employee. Participants included those who had clinical diagnoses made by qualified healthcare professionals, in addition to individuals who believed themselves to have the condition(s) but had not received such clinical confirmation.

Most autistic participants reported that their autism provided benefits to them with respect to policing work (*n =* 43; 81%). Those participants with a clinical diagnosis of autism were significantly more likely (*P* = 0.025) to report their autistic features providing benefits. According to the qualitative data, participants tended to be less direct in terms of describing how their autistic characteristics brought benefits to their policing work. However, they did demonstrate a commitment to the fundamental goals of the role, such as helping victims, ensuring social justice and dealing with criminals, and they saw this as a rewarding aspect of their career choice. Some participants believed that they could make a difference to society because of their personal profile. A previous survey of autistic people conducted by Cope and Remington^30^ similarly described participants reporting a range of strengths they brought to the workplace, including ‘superior creativity, focus, and memory; increased efficiency and personal qualities such as honesty and dedication; and the ability to offer unique autism-specific perspective’.

However, a similar proportion of participants reported that their autistic features provided challenges to them in their policing work (*n =* 59; 89%), with those who had joined the police force prior to 2006 being significantly more likely to report these challenges (*P* = 0.04). The reasons for this difference are unclear; one possible explanation may be that some autistic employees come to realise the full extent of the challenges they experience after prolonged service (e.g. instances where they felt overlooked for promotion). Participants were particularly expressive about how their autistic characteristics underpinned a range of individual, organisational and structural challenges in policing work. Although there are legislative directives that govern organisational responses to health conditions in the workplace, in practice, a range of difficulties can remain. This is in line with the findings of other studies reporting on the need for wholesale changes to reasonable adjustments for autistic people.^29^ These challenges were reported widely by our participants and were arguably exacerbated by a general reluctance to disclose their identity because of stigma, fears around promotion, other people's perspectives and job opportunities, among other reasons. Arguably, the police force (like many other large organisations) relies on neurotypical ways of structuring and managing workloads and careers, which has important implications for individuals’ mental health and levels of stress. This is important in the context of neurodevelopmental conditions, as those with autism or ADHD are at higher risk of the onset of co-occurring mental health conditions,^3,31^ and the challenges faced in the workplace may exacerbate this. For example, Hayward et al^32^ found that workplace stress had a greater impact on well-being for autistic persons. Although reasonable adjustments may ostensibly be a solution, our participants highlighted how these were failing to fulfil their objectives.

Of the autistic participants who viewed themselves as requiring workplace adjustments owing to their autism diagnosis, only 55% (*n* = 22) reported that such adjustments had been made. Previous survey findings from autistic adults suggest that there are numerous barriers to having workplace adjustments acted on, including both social (e.g. stigmatisation, the status of the person requesting adjustments and the impact of adjustments on others) and organisational (e.g. resource availability, the characteristics of management and the status of the organisation) barriers.^13^

With respect to participants with ADHD (*n =* 51), the majority (78%; *n =* 35) of participants believed that their condition provided them with benefits relating to their work, although almost all participants reported experiencing related challenges (98%; *n =* 98%). Participants with ADHD reported liking the fast pace and diversity of police work, in contrast to the structure and routine preferred by autistic participants. Such findings indicate that neurodevelopmental conditions should be perhaps factored into decisions relating to career and/or role choice, as previously suggested by Nadeau.^33^ Such considerations will help determine the best fit between an employee and their role, according to the employee's relative abilities and difficulties, coupled with the demands associated with different occupational roles. As Nadeau^33^ describes, ‘a lifetime of struggling with tasks for which one is ill suited is like a life a life of wearing never wearing shoes that fit’. Thus, although reasonable adjustments should be made in all circumstances, it is likely to be in both employee's and employer's best interests for individuals to work in a role well suited to their relative strengths and difficulties.

Participants with a clinical diagnosis of ADHD were significantly more likely to view themselves as requiring workplace adjustments compared with their self-diagnosed peers (*P =* 0.03). There are multiple possible explanations for this. It could be that the clinically diagnosed individuals on average experienced greater workplace challenges resulting from their symptoms, or indeed that individuals experiencing greater difficulties were more likely to pursue a formal diagnosis, possibly viewing this as an important step in getting any required adjustments acted on.^13^ Of the ADHD participants who had requested adjustments to their role owing to their ADHD diagnosis (51%; *n =* 23), only 35% (*n =* 8) had such requested adjustments made, suggesting that many of the barriers experienced by autistic adults may also exist for their peers with ADHD,^13^ although there may also be ADHD-specific barriers.

With respect to co-occurring mental health conditions, the participant population reported high rates of both anxiety (49%; *n* = 57) and depression (34%; *n =* 40), with similarly high prevalence rates reported for those with a clinical diagnosis of autism (depression: 32%, *n =* 21; anxiety: 47%, *n =* 31) and ADHD (depression: 35%, *n* = 18; anxiety: 45%, *n* = 23). Although these rates are higher than those reported in the general population,^34^ anxiety and depression are both more prevalent among autistic persons^35^ and persons with ADHD^36,37^ who are clinically diagnosed. However, Gullon-Scott and colleagues^38^ reported significantly higher rates of anxiety and depression among police officers compared with the general population; thus, it appears that police work in general may increase one's risk of such conditions.

In summary, when comparing the autistic participants with those with ADHD, the quantitative findings were broadly similar. The majority of participants in both groups reported their conditions providing both benefits and challenges relating to their policing work. Furthermore, the majority of both groups reported needing workplace adjustments, but when such adjustments were requested, they were made only inconsistently for both groups. With respect to the qualitative findings, there did appear to be differences in motivation for a career in policing, with autistic participants expressing an appreciation for structure and routine, whereas those with ADHD reported an appreciation of the energy and fast pace associated with their career choice. However, one must be cautious in the interpretation of such findings, as a substantial proportion of the autistic subgroup had co-occurring ADHD (*n =* 17; 26%) and a substantial proportion of the subgroup with ADHD were also autistic (*n =* 17; 33%).

### Limitations

The data reported here are based on self-reported information from participants, so self-reported clinical diagnoses of autism and/or ADHD were not confirmed via participants’ medical records. Furthermore, although many participants reporting self-diagnosis of autism and/or ADHD (i.e. believing themselves to have either or both conditions in the absence of a clinical diagnosis by a qualified healthcare professional) may have the condition(s) they believe themselves to have, there is uncertainty as to whether they would meet the threshold for clinical diagnosis were they to undergo formal diagnostic assessment. However, it is important to consider the long, often prohibitive waiting times for autism and ADHD clinical assessments in the UK.^39,40^ Furthermore, research evidence suggests that many autistic persons^2,41^ and persons with ADHD^42^ are undiagnosed. Thus, a survey design requiring participants to self-report only clinically confirmed diagnoses of autism and/or ADHD would risk effectively excluding many undiagnosed participants with these conditions.

Another limitation of this study was the lack of participants from minority ethnic groups, as 91% of participants (*n =* 106) reported being of White British or Northern Irish ethnicity. However, this ethnicity distribution does appear to broadly reflect the overall ethnicity distribution of the wider UK police force, which as of March 2020 comprised a 93% White police officer workforce, with only 7% of officers belonging to Black and minority ethnic groups.^43^ By comparison, the general UK population consists of 82% White ethnicity and 18% from all other ethnic groups.^44^

Furthermore, none of the survey items was mandatory. This was felt by the research team to be the most appropriate and ethical approach to the survey, so that participants were not obliged to provide answers where they did not wish to do so. However, this approach inevitably led to responses not being provided by all participants to all items, even when such items were applicable to the participant in question.

Although workplace adjustments were widely requested among participants who were autistic and/or had ADHD, these were frequently not acted on. However, we did not collect data pertaining to the precise adjustments that were requested, and it is possible that some requests were not considered reasonable. Petty et al^45^ have attempted to define what constitutes a reasonable adjustment in the context of autistic employees, and further research could involve data collection relating to the nature of requested adjustments as well as potentially the views of corresponding line managers.

Davies and colleagues,^13^ in investigating the experiences of autistic adults in receiving workplace adjustments, theorised that one might expect to observe different adjustment experiences between those having and lacking a clinical diagnosis, considering the perception that this is associated with a legal right to such workplace adjustments. However, they reported that they were unable to make such comparisons owing to having only a low number of participants self-identifying as autistic. In our study population, for both autism and ADHD, there were no significant differences in workplace adjustments being acted on based on having a clinical diagnosis, although this may be in part a reflection of the relative sizes of these participant subgroups. However, a clinical diagnosis of ADHD was significantly associated with viewing oneself as requiring workplace adjustments to one's role (*P* = 0.03); one possible explanation may be that participants with ADHD experiencing a greater level of functional impairment may be more motivated to pursue a clinical diagnosis.

Finally, although qualitative data were collected, this was done via the online survey rather than interview; the survey relied on textual responses, which tended to be short. A more in-depth qualitative study conducted using an interview-based approach would provide greater richness of detail and further context regarding the subject matter. Furthermore, the opportunity to talk through the issues could provide a mechanism for exploration of the specific issues that were raised in the responses to the questionnaire.

### Clinical implications

Healthcare professionals, including psychiatrists, psychologists and other professionals, have a central role in the assessment and diagnosis of autism and ADHD. The findings of this survey underline the importance of occupational considerations when working with people with these conditions, as well as advocating with employers for reasonable workplace adjustments to be made and providing ongoing support in the workplace (e.g. by psychologists working closely with police force employees). Furthermore, policing work may confer an additional risk of anxiety and depression among police force employees, and it is important to be particularly mindful of the need to also assess for co-occurring mental illness when conducting assessments for neurodevelopmental conditions.

### Policy implications

The findings reported here suggest that UK-based police force employees who are autistic and/or have ADHD frequently have co-occurring mental illness, predominantly anxiety and depression. The findings will be shared with police force employees and stakeholders, with a view to further developing the workplace environment to be more supportive of staff who are autistic and/or have ADHD. This will involve both making reasonable adjustments for any difficulties such individuals may experience in their policing work and making the most of strengths related to their autism and/or ADHD. Indeed, some authors have recently argued in favour of a positive autism approach that focuses on building on talents in the workplace rather than improving weaknesses.^46^ This understanding is especially important in the modern world, as organisations are in a position of needing to retain their workforce, and in the current moment there is a greater spotlight on employee well-being and job satisfaction. Thus, the rewards of any role are instrumental in retention and productivity, and adjustments and recognition of equality and diversity characteristics are centralised.

### Research implications

Further research is required to gain further understanding of the experiences of police force employees who are autistic and/or have ADHD, including those related to specific roles within the organisation, as well as the experiences of those who are also members of ethnic minority groups. In addition, research should consider not just current police force employees who are autistic and/or have ADHD but those who wish to work for the police but have been unsuccessful in their application(s), as autistic adults in particular experience specific barriers to successful recruitment.^19^ Another group of interest for further study would be former police force employees who may have left for reasons related to their neurodevelopmental condition(s). Furthermore, any interventions and/or policy changes to improve the police workplace for employees who are autistic and/or have ADHD should be subjected to rigorous scientific study, to measure their impact as objectively as possible.

Surveys such as that used in this study could also provide a framework for studying adults with such conditions in other occupational groups, as there is a general lack of research evidence pertaining to the workplace experiences of adults who are autistic and/or have ADHD. Collecting such data and ensuring that subsequent findings lead to meaningful policy change is essential to achieve the goal whereby many more such individuals will be able to thrive in their workplace.

## Data Availability

The data that support the findings of this study are available from the corresponding author upon reasonable request.

## Footnotes

a Described on their website as ‘a privately-run group of police officers and staff supporting colleagues affected by autism and other neurodivergent conditions such as dyslexia, dyspraxia and ADHD’.

b Membership count performed closest to the date of the survey being sent out.

c 1980 was the median year of birth within the study population.

d 2006 was the median year of joining the police force within the study population.

e In the survey, participants were asked what their sex was (not their gender), with the following options being available: male, female, transgender, non-binary/non-conforming and prefer not to say. In retrospect, given the options provided, this should have been phrased as instead enquiring about the participant's gender, and as such the term gender has been used both here and in the results tables.
